# Hexamer structure of *Pseudomonas aeruginosa* deoxynucleotide triphosphate triphosphohydrolase and its involvement in catalysis and DNA binding

**DOI:** 10.1063/4.0000995

**Published:** 2025-10-27

**Authors:** Han Byeol Oh, Sung-il Yoon

**Affiliations:** Kangwon National University, Chuncheon, Kangwon, Korea, Republic of

## Abstract

Genetic mutations often have lethal consequences for cells, occurring more frequently when the pool of deoxynucleotide triphosphates (dNTPs) is imbalanced. To keep the dNTP pool balanced, intracellular dNTP levels are tightly regulated through dNTP biosynthesis and degradation pathways. dNTP triphosphohydrolase contributes to dNTP homeostasis and the fidelity of DNA replication by hydrolyzing dNTP into 2'- deoxyribonucleoside and inorganic triphosphate. In this study, we conducted structural analysis to provide insight into the dNTP hydrolysis mechanism used by the dNTP triphosphohydrolase of the opportunistic pathogen *Pseudomonas aeruginosa* (paDT). paDT consists of three domains and assembles into a hexamer as a characteristic quaternary structure of phylogenetic clade A of dNTP triphosphohydrolases. Each subunit of the paDT hexamer accommodates a metal ion in an interdomain dent using a conserved motif composed of histidine and aspartate residues. This observation and our comparative analyses suggest that the interdomain dent functions as the active site of paDT. Interestingly, paDT showed ssDNA-binding ability, presumably via a positively charged patch located in the intersubunit cleft, allowing us to propose that paDT recognizes ssDNA as an allosteric regulator.

